# Genomic Analysis Defines Increased Circulating, Leukemia-Induced Macrophages That Promote Immune Suppression in Mouse Models of FGFR1-Driven Leukemogenesis

**DOI:** 10.3390/cells14191533

**Published:** 2025-09-30

**Authors:** Ting Zhang, Atsuko Matsunaga, Xiaocui Lu, Hui Fang, Nandini Chatterjee, Ahmad Alimadadi, Stephanie F. Mori, Xuexiu Fang, Gavin Wang, Huidong Shi, Litao Zhang, Catherine C. Hedrick, Bo Cheng, Tianxiang Hu, John K. Cowell

**Affiliations:** 1 Georgia Cancer Center, 1410 Laney Walker Blvd., Augusta, GA 30912, USA; tizhang@augusta.edu (T.Z.); adowney@augusta.edu (A.M.); xlu1@augusta.edu (X.L.); hfang@augusta.edu (H.F.); stmori@augusta.edu (S.F.M.); xfang@augusta.edu (X.F.); hshi@augusta.edu (H.S.); 2 Department of Dermatology, Tianjin Academy of Traditional Chinese Medicine Affiliated Hospital, Tianjin 300120, China; zhanglitao@medmail.com.cn; 3 Department of Stomatology, Zhongnan Hospital of Wuhan University, Wuhan 430071, China; chengbo@znhospital.cn; 4 La Jolla Institute for Immunology, La Jolla, CA 92037, USA; nchatterjee@lji.org (N.C.); aalimadadi@lji.org (A.A.); 5 Department of Biology and Psychology, University of Georgia, Athens, GA 30602, USA; gavin.wang@uga.edu; 6 Immunology Center of Georgia, 1410 Laney Walker Blvd., Augusta, GA 30912, USA; lhedrick@augusta.edu

**Keywords:** FGFR1 leukemogenesis, monocytes, macrophages, CyTOF, scRNA-Seq, immune suppression

## Abstract

The development of FGFR1-driven stem cell leukemia and lymphoma syndrome (SCLL) in mouse models is accompanied by an increase in highly heterogenous myeloid derived suppressor cells (MDSCs), which promote immune evasion. To dissect this heterogeneity, we used a combination of CyTOF and scRNA-Seq to define the phenotypes and genotypes of these MDSCs. CyTOF demonstrated increased levels of circulating macrophages in the peripheral blood of leukemic mice, and flow cytometry demonstrated that these macrophages were derived from Ly6C^Hi^ M-MDSC as well as the Ly6C^Int^ and Ly6C^Low^ monocytic populations. Consistently, scRNA-Seq analysis demonstrated the accumulation of non-classical monocytes (ncMono) during leukemia progression, which also express macrophage markers. These leukemia-induced macrophages show continuous transcriptional reprogramming during leukemia progression, with the upregulation of cellular stress response genes *Hspa1a* and *Hspa1b* and inflammation-related gene *Nfkbia*. Trajectory analysis revealed a transition from classical monocytes (cMono) to ncMono, and potential genes orchestrating this transition process have been identified. Furthermore, T-cell suppression assays demonstrated the immune suppressive abilities of leukemia-induced circulatory macrophages. Targeting these macrophages with the GW2580 CSF1R inhibitor leads to restored immune surveillance and improved survival. Overall, we demonstrate that circulating macrophages are responsible, at least in part, for the immune suppression in SCLL leukemia models, and targeting macrophages in this system improves the survival of leukemic mice.

## 1. Background

Hematologic malignancies associated with *FGFR1* abnormalities typically present as a myeloproliferative neoplasm but then develop into heterogeneous forms of leukemia including acute myeloid leukemia (AML), T- or B-lineage lymphoblastic leukemia/lymphoma, and even mixed phenotype acute leukemia [1]. The hallmark of the resulting stem cell leukemia/lymphoma syndrome (SCLL) is the presence of chimeric FGFR1 genes formed as the result of chromosome translocations, which bring a dimerization motif from the partner chromosome in juxtaposition with the FGFR1 kinase domain [2]. We have developed murine models of SCLL, which are representative of human disease [3,4,5] and have demonstrated specific FGFR1-directed genetic changes that drive leukemogenesis [6,7,8]. In some cases, these observations have identified therapeutic candidates which, when targeted pharmacologically, can suppress leukemogenesis in these models. The homogeneous nature of these leukemia models provides an opportunity to characterize the genetics of FGFR1-driven tumorigenesis and investigate their influence on host immune surveillance and the underlying mechanisms.

CyTOF and single-cell mRNA sequencing (scRNA-Seq) are both powerful techniques to characterize the tumor immune microenvironment. CyTOF uses heavy metal labeled antibodies against cell surface markers, which are used to define their distribution on individual cells in the sample [9]. Unlike traditional flow cytometry, which can only analyze relatively limited numbers of markers in a single experiment, CyTOF can simultaneously identify more than 100 different markers. When CyTOF is used in combination with scRNA-Seq, the reprogrammed gene expression in cells in the immune system can be superimposed on cell phenotypes identified through cell surface marker expression. These studies provide a more complete characterization of how the immune cell microenvironment responds to the presence of cancer cells. To date, the majority of studies in this regard have been performed in solid tumors, with few investigations reported for leukemias, which have a distinct pathobiology compared with solid tumors. Although microenvironment studies have been reported in leukemias, these have been limited to flow cytometric analyses using a limited subset of cell surface markers [10,11].

To provide a better characterization of leukemia-induced changes in the SCLL immune microenvironment, we investigated the immune cell composition in the peripheral blood (PB) from SCLL mice using CyTOF in combination with scRNA-Seq. CyTOF analysis demonstrates increased levels of macrophages in the peripheral circulation of leukemic mice, which was further confirmed by flow cytometry and electron microscopy (EM). Both the non-classical monocytes (ncMonos) and classical monocytes (cMonos) contribute to these circulatory macrophages [12]. Using scRNA-Seq, the gene signature associated with these leukemia-induced macrophage-like ncMonos has been identified, as well as transcriptional reprogramming in these macrophages during leukemia progression. Trajectory assay suggests a transition from cMonos to ncMonos in leukemic mice and the modulatory genes during this transition have been defined. T-cell suppression assays in vitro and macrophage depletion in vivo confirmed a direct role for these leukemia-induced macrophages in suppression of anti-leukemia immunity.

## 2. Methods

### 2.1. In Vivo Studies

The 0.1 × 10^6^ SCLL cells were engrafted into 6–8-week-old, female Balb/C mice, as described previously [13], and PB was collected from the tail vein for flow cytometry analysis. For macrophage depletion, GW2580 (MedChemExpress, Monmouth Junction, NJ, USA, #HY-10917) was administrated by IP daily at 80 mg/kg, starting 5 days after SCLL cell engraftment. All animal experiments were performed under an approved protocol from the Augusta University Institutional Animal Care and Use Committee.

### 2.2. CyTOF Analysis of Peripheral Blood Samples

Individual cells from PB samples were stained with a panel of 39 individual antibodies (Supplementary Table S1) and analyzed using a CyTOF XT Mass Cytometer (Standard Biotools, San Francisco, CA, USA). Sample processing was in triplicate and data collection and analysis was performed as previously described [14]. Data files were normalized using the bead-based Normalizer [15], which was then analyzed using the cloud-based OMIQ platform (omiq.ai, Dotmatics). Dimensionality reduction was performed using the Uniform Manifold Approximation and Projection (UMAP) algorithm [16,17], and hierarchical clustering was performed using the FlowSOM algorithm Trajectory Inference using the Wishbone algorithm [18].

### 2.3. Flow Cytometry Analysis

PB was collected from the tail vein, and red blood cell lysis was performed to enrich the leukocytes. Flow antibodies used in this study were CD4-APC (Biolegend, San Diego, CA, USA, #100412), CD8α-PE/Cy7 (Biolegend, #100722), Ly6C-PE (Biolegend, #128008), CD11b-PerCP/Cy5.5 (Biolegend, #101228), Ly6G-APC/Cy7 (Biolegend, #127624), F4/80-APC/Cy7 (Biolegend, #123118), and CD135-BV421 (Biolegend, #135315). The NovoCyte Quanteon Flow Cytometer System was used for data collection, and analysis was performed using FlowJo™ v10.10.

### 2.4. T-Cell Proliferation Assay

CD11b+F4/80+ macrophages were sorted from the bone marrow of naïve C57BL6 mice and the peripheral blood of leukemic mice. Spleen-derived CD4+ T cells were isolated using the MojoSort™ Mouse CD4 T Cell Isolation Kit (Biolegend, #480033) from the naïve C57BL6 mice. The isolated CD4+ T cells were first stained using the CFSE Cell Division Tracker Kit (Biolegend, #423801) and then co-cultured with macrophages for 3 days at 1:1 or 2:1 ratio in the presence of anti-CD3/anti-CD28 Dynabeads (Sigma-Aldrich, St Louis, MO, USA, #11452D). T-cell proliferation was then analyzed using flow cytometry for CFSE by gating on the CD4+ population.

### 2.5. Electron Microscopy

Isolated cells were postfixed in 2% osmium tetroxide in sodium cacodylate (NaCAC) buffer, stained and blocked with 2% uranyl acetate, dehydrated using a graded ethanol series, and embedded in Epon 812 Araldite resin mixture (Electron Microscopy Sciences, Hartfield, PA, USA). Thin sections were cut with a diamond knife using a Leica EM UC7 ultramicrotome (Leica Microsystems, Inc., Bannockburn, IL, USA), collected on copper grids, and stained with 2% uranyl acetate and lead citrate. Cells were observed in a JEM 1230 transmission electron microscope (JEOL USA Inc., Peabody, MA, USA) at 110 kV and imaged with an UltraScan 4000 CCD Camera (Pleasanton, CA, USA).

### 2.6. RNA-Seq and Quantitative Real-Time PCR

The qRT-PCR and RNA-Seq analysis was performed as previously described [3]. The sequences of primers used are described in Supplementary Table S2.

### 2.7. Single-Cell RNA Genomic Analysis

Preparation, sequencing, and bioinformatics analysis of PB samples was carried out as described previously [19].

### 2.8. Statistical Methods

The Student’s *t* test was performed for comparison between two groups, whereas one-way ANOVA multiple comparison was used for comparison of three or more groups. ns represents not significant, * *p* < 0.05, ** *p*< 0.01, *** *p* < 0.001, and **** *p* < 0.0001.

## 3. Results

### 3.1. CyTOF Analysis Identifies Leukemia-Induced Macrophage Populations

In our previous study, flow cytometry analysis identified leukemia-associated polymorphonuclear MDSC (PMN-MDSC) and monocytic MDSC (M-MDSC) based on the expression of cell surface markers CD11b, Ly6C, and Ly6G [13]. This approach, however, only provides a limited view of immune cell composition because of the limited detection capability. CyTOF, however, can provide a high-dimensional and unbiased analysis of the immune cell composition, which can define individual disease-associated cell populations. To further examine the effects of SCLL cells on the global immune microenvironment, we engrafted 0.1 × 10^6^ BBC2 cells into three individual mice and sacrificed them after 14 days. The BBC2 cell line was established from primary leukemia cells arising from stem cells transduced with the BCR-FGFR1 chimeric kinase that gives rise to a pro-B-cell-like leukemia [4]. This cell line model represents the acute phase of this leukemia with mice succumbing within 15–20 days [4]. The value of this in vivo system lies in the fact that the tumor cells co-express GFP from the transducing vector, allowing leukemic cells to be distinguished from normal cells in the peripheral circulation.

**Figure 1:**
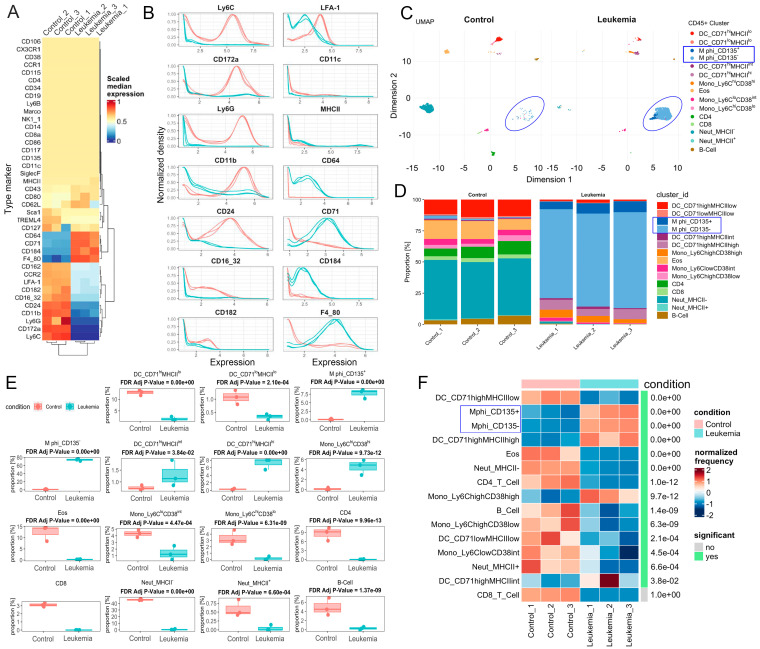
CyTOF identifies leukemia-induced macrophages in the peripheral circulation in SCLL model. Heat map (**A**) of the relative expression levels of 39 CyTOF markers in bulk, frozen peripheral blood (PB) samples from three individual naive mice and three leukemia bearing mice. Differential expression in the two cohorts identifies surface markers that are significantly upregulated and downregulated in leukemic mice compared with naive mice. The scaled expression levels of the most highly dysregulated individual markers from the 3 individual mice in naive control (red traces) and leukemic (blue traces) mice are shown in (**B**). Using the UMAP algorithm, the individual cells are categorized into fifteen clusters based on their composite surface protein expression pattern, where the CD135− macrophage cell population (circled) dominates the PB from the leukemic mice (**C**). The identities of the cells in the fifteen individual clusters were annotated based on the individual expression profiles. The proportions for different clusters in each of the three different mice from naive mice (**left**) and leukemic mice (**right**) were plotted in stacked bar graphs (**D**), revealing consistent differences in individual cell types. When this data is compiled into box plot comparisons (**E**), the significances in changes in different clusters are displayed. The normalized frequencies for these clusters are plotted in the heatmap (**F**). There are significant changes between control and leukemic mice for all cell types, except CD8+ T cells. The blue circles and boxes highlight the predominant macrophages identified in the peripheral circulation of leukemic mice.

Frozen, GFP-negative PB leukocytes were then prepared and shipped for off-site CyTOF analysis, together with samples from three naive mice as controls. Only CD45+ cells were included in the analysis, which is a broad marker of hematopoietic cells. From the PB leukocytes, 1194–1391 cells from each naive mouse were analyzed, as well as 1400 cells from each leukemic mouse. The expression of 39 individual immune cell surface markers was then characterized in these cells. As shown in the heatmap in Figure 1A, in a comparison between CD45+ PB cells from naive and leukemic mice, there was a consistent increase in the expression levels of markers CD64, CD71, CD184, and F4/80 in the three leukemia-bearing mice. There was also a decrease in expression levels of CD24, CD11b, Ly6G, CD172a, and Ly6C and, to a lesser extent, CD162, CCR2, LFA-1, CD182, and CD16/32. The surface protein levels for each of these markers in the three individual mice from both cohorts is shown in Figure 1B. Using the Uniform Manifold Approximation and Projection (UMAP) algorithm, cells were grouped into 15 individual clusters based on the expression of the 39 markers (Figure 1C, Supplementary Figure S1).

Cluster annotations were assigned based on their relative expression of established cell-type markers (Supplementary Figure S1) and cross referenced to published studies of these cell types. The dendritic cells are defined by the expression of CD11b, TREML4, and CD11c, and are sub-grouped by both high and low CD71 expression as well as high, intermediate, and low MHCII expression. Macrophages are defined by the expression of CD11b, TREML4, F4/80, and Marco and sub-grouped into CD135+ and CD135−. Monocytes are defined by the expression of CD11b and are sub-grouped by both high and low Ly6C expression as well as high, intermediate, and low CD38 expression. Neutrophils are classified by the expression of CD11b, Ly6C, and Ly6G and are divided into MHCII+ and MHCII− subtypes. Eosinophils are defined by CD11b and Siglec-F. B cells are classified by CD19, CD4 T cells by CD4, and CD8 T cells by CD8.

When changes in the proportion of cells in each of these clusters were compared between naive and leukemic mice (Figure 1D,E), besides often small but significant differences between individual cell types, major changes were seen in the CD135− macrophages, which constituted ~75% of the cells in the processed PB sample, as well as 8% of the CD135+ macrophages. Thus, in this analysis, 83% of the total cells in the samples from leukemic mice were macrophages. In the PB from normal mice, however, there were virtually no CD135+ or CD135− macrophages. Levels of CD4+ and CD8+ T cells were reduced from 9% and 3%, respectively, in the PB of naive mice to virtually no T cells in the PB of leukemic mice. In the PB from the naive mice, the levels of MHCII- neutrophils and CD71^hi^ MHCII^lo^ dendritic cells (DCs) were reduced from 45% and 14% of the total, respectively, to almost zero in the leukemic mice. Relative normalized frequencies for all clusters are shown in Figure 1F, where the changes were significant in 14 out of 15 clusters, with the exception of CD8+ T cells.

**Figure 2:**
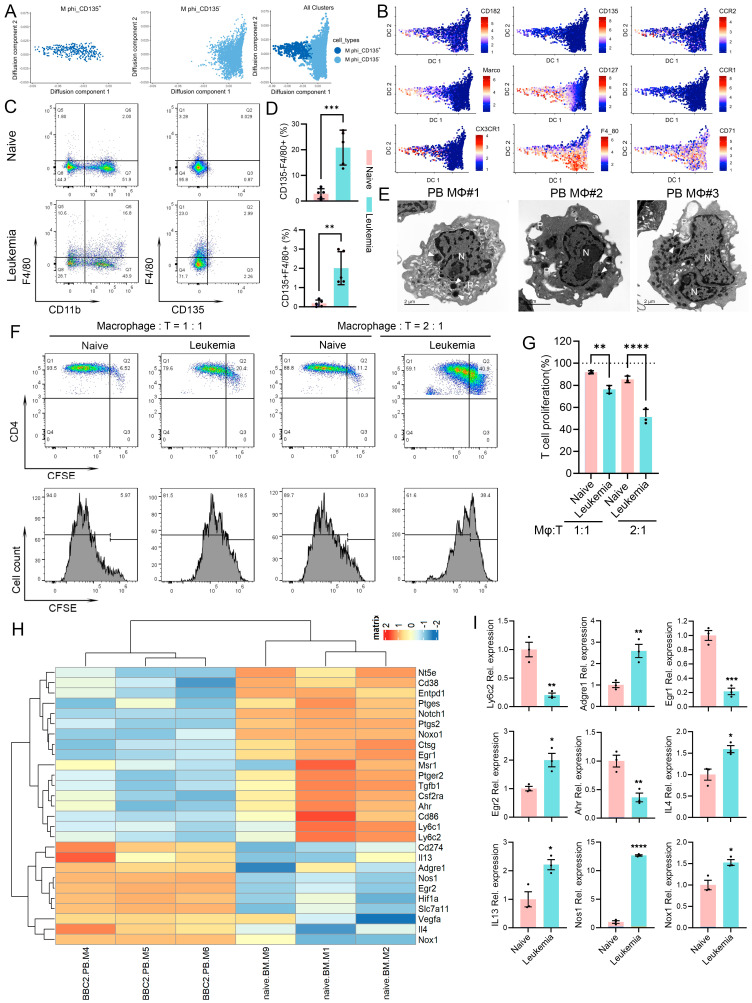
Leukemia-induced circulating macrophages perform an immune suppression function. Diffusion maps (**A**) define the CD135+ (**left**) and CD135− (**center**) macrophage clusters, which, when combined (**right**), demonstrate a contiguous, branching profile. Expression levels for individual genes within the diffusion map demonstrate specific, high-level expression of indicated genes in the CD135+ macrophages but decreased expression in the CD135− population (**B**). Flow cytometry shows increased levels of both the CD135− and CD135+ macrophages in the PB of leukemic mice compared with naive mice (**C**). Quantitation of indicated macrophages (**D**) in leukemic mice compared with naive mice (*n* = 3) are shown in (**D**). The identity of F4/80+ macrophages was confirmed using electron microscopy (**E**) demonstrating large nuclei (N), phagosomes (P), and lysosomes (arrows). T cells (CD4+) were co-cultured with macrophages (MΦ) from naive mice mixed at a 1:1 ratio, showing high levels of proliferation (91.94%). When CD4+ T cells were cultured with macrophages derived from leukemic mice and mixed at the same ratio, T cell levels were reduced to 76.4%. When T cells were mixed with macrophages at a 1:2 ratio, T-cell proliferation activity was further reduced to 51.27% (**F**,**G**). A heatmap plot comparing the expression levels from RNA-Seq data for selected immune suppression-related genes between control macrophage from naive mice and circulatory macrophages from leukemic mice (**H**). qRT-PCR detection of the expression levels of indicated genes between macrophages from naive and leukemia mice (**I**). * *p* < 0.05, ** *p*< 0.01, *** *p* < 0.001, and **** *p* < 0.0001.

It should be noted that the PB samples in this study were frozen prior to submission to the CyTOF facility, and it has been shown that this results in the loss of most of the fragile neutrophils in the samples [20]. The CyTOF analysis, therefore, provides valuable information that is largely restricted to the mononuclear cells in the analysis and the relative abundances of the same clusters between naive and leukemic mice. With this caveat, macrophage clusters 3 (CD135+) and 4 (CD135−) represented the major differences between the naive and leukemic samples (Figure 1C). To resolve the connections of these tightly associated clusters, we performed a diffusion map analysis (Figure 2A), which revealed that the two subgroups of cells appeared as a continuous branching profile rather than two distinct clusters. These two clusters shared similar expression levels for most of the markers detected, such as F4/80 and CD71(Figure 2B, Supplementary Figure S1). These diffusion maps suggest a differentiation from the CD135+ progenitor cells into the more mature CD135- macrophages in a differentiation continuum. The markers that can be used to distinguish the CD135+ progenitor cells are CX3CR1, CCR1, CD127, Marco, CCR2, CD135, and CD182, most of which are involved in macrophage activation and function. Diffusion map analysis of the expression levels of these markers in the CD135+ macrophages demonstrate a higher-level expression of CD127, Marco, CX3CR1, and CCR2 (Figure 2B). Notably, a high-level expression of F4/80 and CD71 is detected in both macrophage populations.

### 3.2. Leukemia-Induced Immunosuppressive Macrophages in Peripheral Blood

In solid tumors, macrophages are confined to the tumor mass where they differentiate from infiltrating monocytes and are rarely seen in the peripheral blood. Our CyTOF analysis of PB revealed a predominant presence of macrophages in leukemic mice. To confirm this observation, flow cytometry was used to detect the presence of macrophages in the peripheral circulation. F4/80+CD135− macrophages in naive mice represent only 2.83% of cells in the PB, which increases to 20.84% in the leukemic mice, while the F4/80+CD135+ macrophages comprise 0.18% of leukocytes in naive mice, which increased to 2.01% in leukemia mice (Figure 2C,D). To confirm the identity of these leukemia-induced macrophages, we sorted Cd11b+F4/80+ cells from the PB of leukemic mice and performed electron microscopy. As shown in Figure 2E, these cells showed typical structures of macrophages with enlarged nuclei and abundant phagosomes and lysosomes.

To determine whether the macrophages that accumulate in the leukemic mice have a functional effect on T cells, we performed T-cell suppression assays. CD4+ cells were isolated from the spleens of naive mice and co-cultured with sorted macrophages from leukemic mice at D14, based on the expression of CD11b and F4/80. For the naive mice, CD11b+F4/80+ macrophages were isolated from the bone marrow and, when cultured with CD4+ T-cells at a 1:1 ratio, 85% of the CD4+ T cells showed cell proliferation (Figure 2F,G). When macrophages from the PB of leukemic mice were co-cultured with CD4+ T-cells and mixed in a 1:1 ratio, proliferating T cells were reduced to 71%. When the T-cell/macrophage ratio was increased to 1:2, there was only a slight decrease in T-cell proliferation in the naive group but a far more significant reduction in T-cell proliferation in the co-culture with macrophages from leukemic mice (Figure 2F,G). RNA-Seq analysis of these macrophages from naive and leukemic mice revealed an increased expression of macrophage markers *Adgre1* (encoding F4/80) and *Egr2*, immune suppression-related molecules *Cd274*, *Nos1*, *Hif1a*, *Vegfa* and *Nox1*, as well as cytokines for M2 macrophage polarization *Il4*, and *Il13* (Figure 2H). In contrast, M1 macrophage-related markers including *Egr1*, *Msr1*, *Ahr*, *Cd86*, *Ly6c1*, and *Ly6c2* show a reduced expression in leukemia-induced macrophages. The relative expression levels of these markers in macrophages were further confirmed by qRT-PCR (Figure 2I).

### 3.3. M-MDSC Partially Contribute to Leukemia-Induced Macrophages in Peripheral Blood

**Figure 3:**
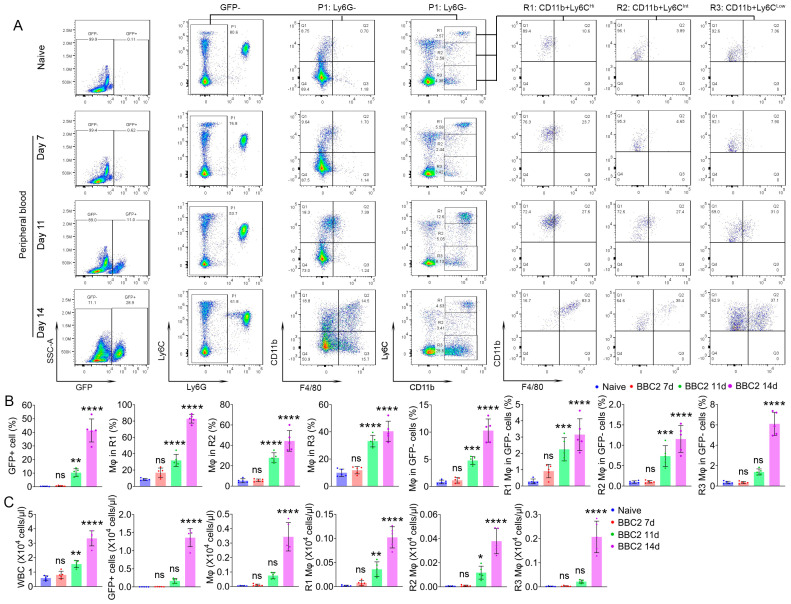
Leukemia-induced reprogramming of monocytes into macrophages in SCLL model. Flow cytometric analysis (**A**) of GFP-expressing leukemic cells shows a progressive increase over the 14-day time course. Gating on the GFP−Ly6G− cells shows increased levels of the CD11b+F4/80+ macrophages in the peripheral blood samples during leukemia progression. CD11b+ myeloid cells in the GFP-Ly6G- population were further sub-grouped into Ly6C^Hi^ (M-MDSC), Ly6C^Int^, and Ly6C^Low^ cells. In all three clusters, there is a continuously increased presence of CD11b+F4/80+ macrophages during leukemia progression, with the highest purity in the Ly6C^Hi^ (M-MDSC). The relative percentages of indicated cells against the specific clusters or total GFP− cells are shown in (**B**), and cell counts of indicated cells are shown in (**C**). Hi, high; Int, intermediate. * *p* < 0.05, ** *p*< 0.01, *** *p* < 0.001, and **** *p* < 0.0001.

Our previous report showed an increase in MDSC during SCLL progression in the peripheral circulation [13]. Here, the CyTOF and flow cytometry analysis revealed a significant presence of macrophages in the PB. There are reports that indicate M-MDSC can further differentiate into tumor-associated macrophages during cancer development [21,22]. We therefore performed flow cytometry to investigate the connection between M-MDSC and macrophages in our leukemia model. The level of GFP+ leukemia cells increases over 14 days post-engraftment. When we gated GFP− cells and then focused on the Ly6G− population, there was a significant increase in CD11b+F4/80+ macrophages during leukemia progression (Figure 3A,B). To determine which subgroup is giving rise to these macrophages, we parsed the Ly6G−CD11b+ cells based on the Ly6C expression levels and determined the presence of CD11b+F4/80+ cells during leukemogenesis. In the Ly6C^Hi^ group, the relative percentage of macrophages increased from 8.48% in naive mice to 82.69% by day 14 in mice with advanced leukemia. The relative percentage of macrophages increased from 5.30% to 44.33% in the Ly6C^Int^ group and from 9.93% to 40.45% in the Ly6C^Low^ group (Figure 3A,B). These observations suggest that macrophages arise from all subgroups of Ly6G−CD11b+ cells in this model. When the percentages of macrophages from these three subgroups are normalized to total GFP− cells, the most predominant contribution comes from the CD11b+Ly6C^Low^ subpopulation at day 14, which consisted of 6.10% of the total GFP− cells, with 3.15% and 1.15% from the Ly6C^Hi^ group and Ly6C^Int^ group, respectively (Figure 3B). The changes in percentages in the different subgroups of cells were further confirmed by cell counts (Figure 3C).

### 3.4. Molecular Characterization of Leukemia-Induced Monocytes

**Figure 4:**
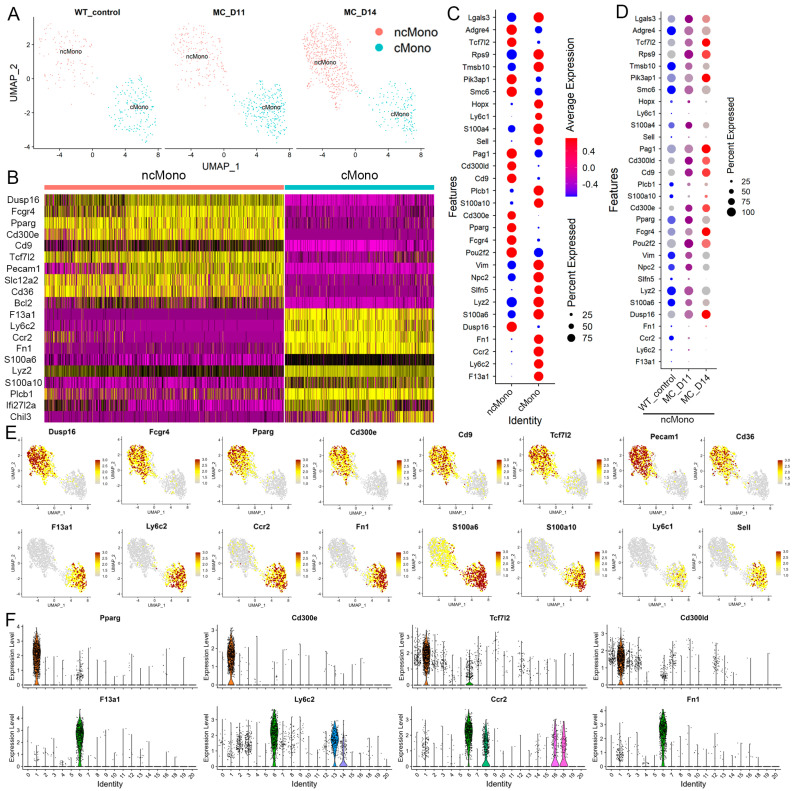
Molecular characterization of leukemia-induced macrophages. The UMAP plot of cMonos and ncMonos shows discrete clusters that are consistent between PB samples from naive and leukemic mice at day 11 and day 14 post-leukemia cell inoculation, showing a dramatic increase in levels of ncMonos (**A**). The 10 most significant genes identified in cMono and ncMono from an analysis of the monocytes from all samples are shown in the heatmap in (**B**) clearly defining the two different populations of monocytes. A dot plot of the expression of the 30 most significant marker genes from ncMonos is shown in (**C**). When the expression levels of the same 30 genes are compared in ncMonos during disease progression, continuous changes in expression are seen for most genes (**D**). Feature plots (**E**) clearly show the exclusive expression of representative genes in the two different monocyte clusters. Violin plots (**F**) across the entire cluster spectrum from PB show the specific expression in ncMonos (cluster 1, above) and cMonos (cluster 6, below) for representative genes.

While CyTOF and conventional flow cytometry can efficiently monitor the dynamic changes in most immune cells in the leukemic microenvironment during leukemia progression based on cell surface marker expression, they do not provide insights into underlying genetic changes in these cells that might directly correlate with their function. Therefore, to address this issue, we performed a, scRNA-Seq analysis using PB leukocytes from mice engrafted with BBC2 SCLL cells. Previous studies [19] had identified the leukemia-induced neutrophil populations in this model but, since the CyTOF analysis showed such dramatic increases in macrophages, here we focused on the changes occurring in monocyte clusters in leukemic mice. Sub-grouped monocytes from the PB leukocytes of naive mice and leukemic mice after 11 days (D11) and 14 days (D14) were analyzed (Figure 4A). Cluster identity predictor (CIPR) analysis defined two distinct subgroups of monocytes, cMono and ncMono, based on the global gene expression profiles of these cells. A heatmap of the 10 most statistically relevant genes identified in the corresponding clusters are shown in Figure 4B. As shown in the UMAP (Figure 4A), only the ncMonos showed progressive increases during leukemia progression whereas, at D14, they constituted 19.5% of total leukocytes compared with 3.2% in naive mice.

A dot plot of the 30 most differentially expressed genes between the two monocyte subgroups is shown in Figure 4C and Supplementary Table S3. Genes that define the ncMonos include *Adgre4*, *Tcf7l2*, *CD300ld*, *Cd9*, *Cd300e*, *Pparg*, *Fcgr4*, *Pou2f2*, and *Dusp16*. Genes defining the cMonos include *Hopx*, *Ly6c1*, *S100a4*, *Sell*, *Plcb1*, *S100a10*, *Slfn5*, *Fn1*, *Ccr2*, *Ly6c2*, and *F13a1*. When the changes in expression levels of the 30 most significant marker genes for the ncMonos were analyzed during leukemia progression, in many cases, the upregulated genes in the leukemic mice showed a progressive increase during the course of the disease (Figure 4D). Feature map views of expression levels for selected genes superimposed on the UMAP plots clearly define their cluster-specific expression patterns (Figure 4E). The specific or predominant expression of indicated genes in the cMono (cluster 6) or ncMono (cluster 1) are further confirmed by violin plots displaying gene expression levels throughout the entire spectrum of cell clusters in the PB leukocytes (Figure 4F). Noticeably, some macrophage marker genes also show an exclusive or predominant expression in the ncMono, including *Adgre1*, *Adgre4*, *Cd9*, *Pparg*, and *Fcgr4*, which indicates a macrophage identity. Therefore, scRNA-Seq data also confirmed the presence of leukemia-induced ncMono/macrophages in our SCLL model.

### 3.5. Leukemia-Induced Macrophage-like ncMonos Undergo Global Transcriptional Reprogramming

**Figure 5:**
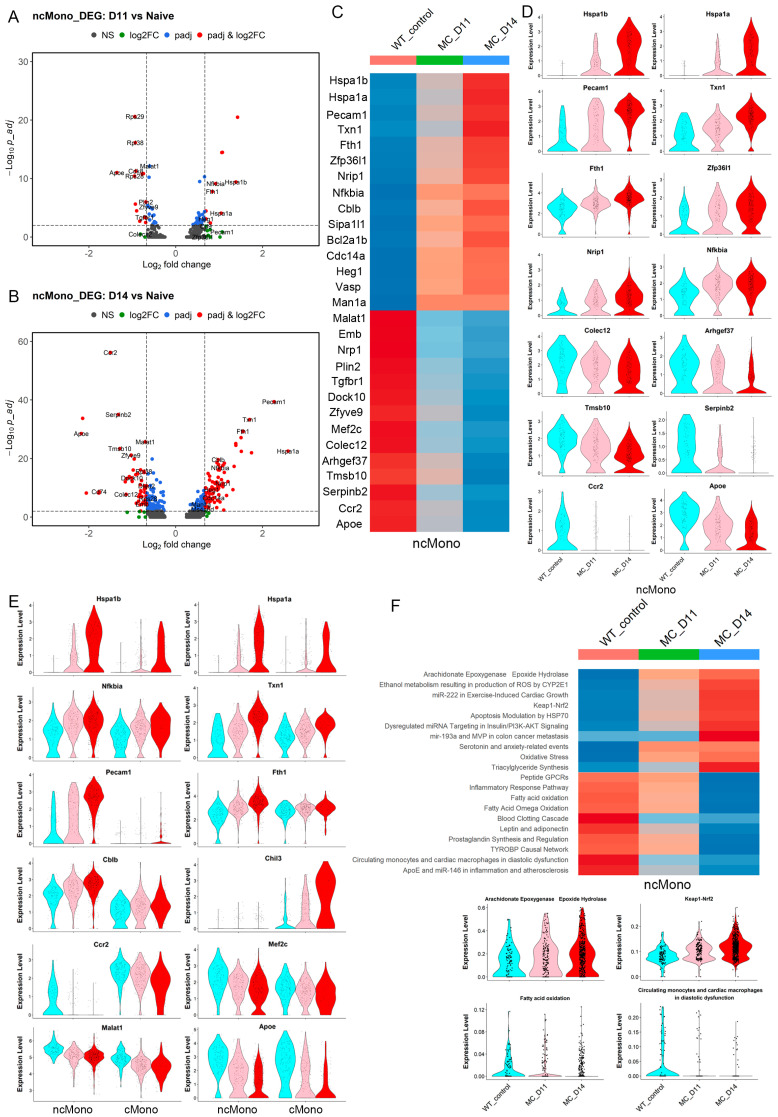
Transcriptional reprogramming in leukemia-induced macrophages during leukemia progression. Volcano plots show the differentially expressed genes (DEGs) in ncMonos at D11 (**A**) and D14 (**B**) compared with naive counterparts, with more DEGs identified in late leukemogenesis. A heatmap plot showing the continuous increase or decrease in the 30 most significant DEGs identified in ncMono (**C**). These expression patterns for the most highly upregulated and downregulated genes are shown in the violin plots in (**D**). A comparison of expression levels of indicated genes between ncMonos and cMonos (**E**) shows similar expression patterns between the two subgroups. A heatmap plot shows the 10 most highly upregulated and downregulated Wikipathways in the ncMonos during leukemia progression ((**F**), above), which are confirmed in the violin plots for selected pathways ((**F**), below).

scRNA-Seq provides an opportunity to define transcriptional reprogramming in different subpopulations of monocytes in response to the presence of leukemia by comparing the same populations at different stages of leukemia. To investigate ncMono-specific gene expression changes during leukemia progression, we created volcano plots which show an emerging trend of transcriptional reprogramming in the early development of leukemia at D11, compared with the naive mice (Figure 5A). These changes become more extensive and pronounced at D14, with more genes showing larger and more significant changes in expression levels (Figure 5B). These genes, therefore, appear to be responding to the presence of the leukemic cells, which is further emphasized in the heatmap in Figure 5C, showing the 29 significant and continuous up- or downregulated genes in leukemia-bearing mice compared with naive mice. While most genes showed quantitative changes in expression levels, heat shock proteins *Hsp1a* and *1b* (encoding HSP70A, B) compared with that of naive mice (Figure 5D). In the cMono cluster, there were fewer genes showing expression level changes, with only 10 upregulated genes and 5 downregulated genes (Supplementary Figure S2). While the two subgroups of monocytes were defined based on differential expression profiles, some genes identified in ncMono showed similar patterns of expression changes seen in cMono during leukemia progression (Figure 5E). For example, *Hspa1a* and *Hspa1b* are upregulated, while *Ccr2* and *Apoe* are downregulated in both the ncMono and cMono.

AUCell analysis, which defines the activities of different molecular pathways within the cell clusters, again revealed transcriptional reprogramming in ncMonos (Figure 5F), where pathways, such as Arachidonate Epoxygenase Epoxide Hydrolase (anti-inflammatory effect through Nfkb [23]), Keap1-Nrf2, and Triacylglyceride Synthesis (involved in redox, metabolism reprogramming [24], and microenvironment remodeling [25]), showed progressive enrichment during leukemogenesis. Pathways involved in inflammatory responses, such as the TYROPB causal network in microglia and circulating monocytes and cardiac macrophages, were progressively downregulated (Figure 5F). When the same analysis was performed on the cMonos (Supplementary Figure S3), while there were some discrete pathway changes compared with ncMonos, several upregulated pathways were shared between the two clusters, such as the epoxegenase, Keap1-Nrf2, triglyceride synthesis, and fatty acid oxidation, and the monocytes in diastolic dysfunction were downregulated in both groups (Supplementary Figure S3).

### 3.6. Trajectory Analysis Reveals a Transition from cMono to Macrophage-like ncMono

**Figure 6:**
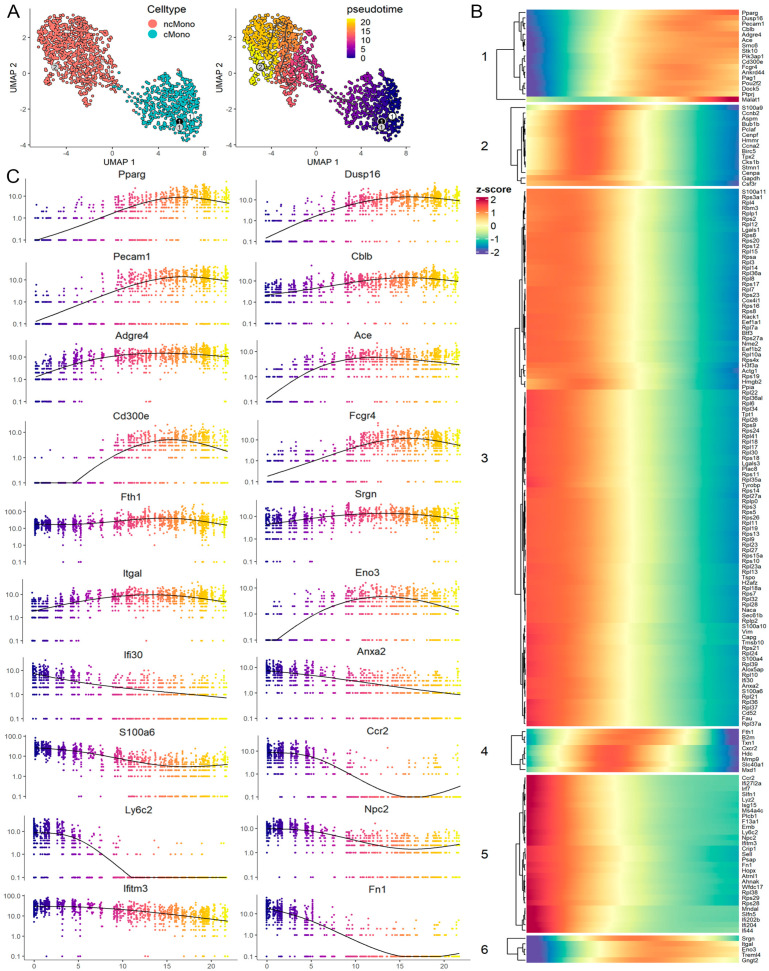
Trajectory analysis of the potential transition path of leukemia-induced macrophages. UMAP plots of the 2 monocytes clusters ((**A**), left) show discrete clusters with a small bridge between them. Pseudo-time analysis ((**A**), right) shows the differentiation path from cMonos through the bridge to the ncMonos. The identified module genes accompanying these transitions are shown in the heat maps in (**B**), with identification of 6 subgroups depending on the pattern of gene expression changes. Analysis of the expression level of specific genes in individual cells using RSS are presented in (**C**), with the color coding defining the timing of expression across the pseudo-time continuum.

To understand the development of monocytes in the PB during leukemogenesis, we performed a trajectory analysis, which seeks to order cells along a pseudo-time based on their gene expression profiles. In the typical UMAP analysis, discrete clusters for the cMono and ncMono were connected by a small bridge (Figure 6A, left). Since ncMonos are induced during leukemia progression, it has been suggested that cMonos give rise to ncMonos [26,27]; the cMonos are assigned as the initiation point for the trajectory analysis. At a branch point within this group, the differentiation continuum reaches across the bridge through four different statuses of ncMonos, terminating in the final subgroup at the most distal end (Figure 6A, right). The expression pattern of potential modulatory genes associated with this transition is shown in Figure 6B, which defines six different dynamic expression patterns. Groups 1 and 6 highlight genes showing a progressively increased expression as differentiation proceeds. The majority of genes, however, show progressive downregulation (groups 2, 3, and 5) at different levels. The genes shown in group 4 show a transient activation during differentiation. The activation of expression (on a logscale) is shown in more detail in Figure 6C, where genes such as *Pparg*, *Pecam1*, and *Dusp16* become progressively more activated through the continuum. Other genes such as *Ccr2*, *Ly6c2*, and *Fn1* are inactivated. There is also a subgroup of genes (e.g., *Mmp9*, *Cxcr2*, and *Txn1*) which are transiently activated during the progression and then inactivated. Importantly, the transition from cMonos to ncMonos shows the progressive upregulation of macrophage and ncMono markers *Adgre1* (F4/80), *Pparg*, and *Nr4a1*, demonstrating that these cells give rise to the macrophages seen in the PB of the leukemic mice (Supplementary Figure S4).

### 3.7. Targeting Leukemia-Induced Macrophages Can Impair Immunosuppression and Improve Survival

**Figure 7:**
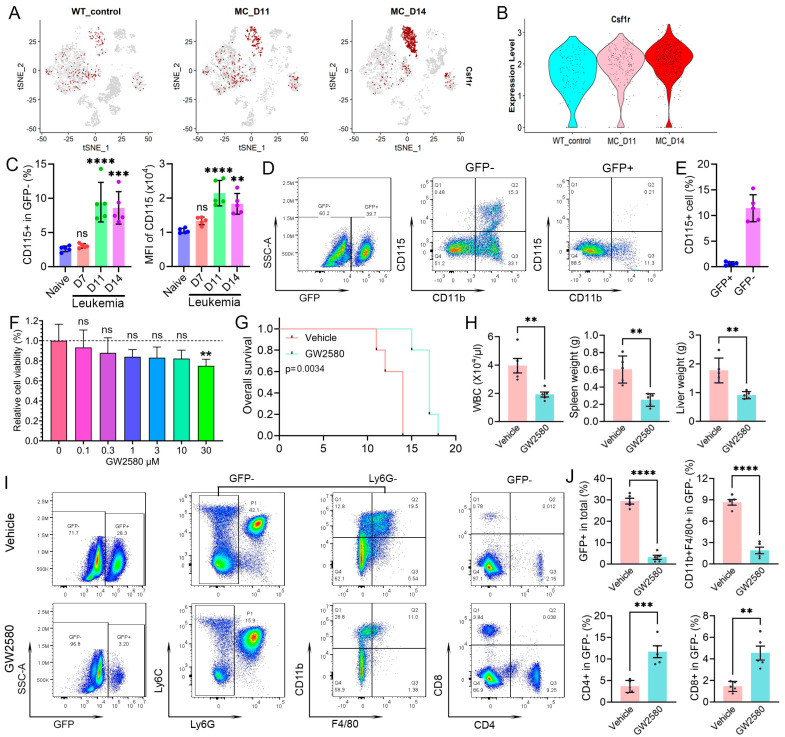
Leukemia-induced macrophages contribute to leukemia progression in a mouse model. Feature plots for *Csf1r* during leukemogenesis are shown in (**A**) and expression levels for *Csf1r* are shown in the violin plots in (**B**). The increased percentages of CD115+ cells in the peripheral circulation and increased levels of CD115 expression were confirmed by flow cytometry (**C**). Flow cytometry demonstrates that the GFP+ leukemia cells in vivo do not express CD115 (**D**), as quantified in (**E**). Treatment of BBC2 leukemic cells with GW2580 at increasing concentrations (**F**) demonstrates limited reduction in cell viability only at high concentrations. When mice engrafted with leukemia cells were treated with the GW2580 CSF1R inhibitor, there was a significant increase in survival (**G**), which is associated with reduced levels of white blood cell counts and decreased spleen and liver weights (**H**). Flow cytometry analysis showed that the GW2580 treatment led to reduced CD11b+F4/80+ macrophages in the GFP-Ly6G- cell population, accompanied by a decrease in GFP+ leukemia cells and an increase in both CD4 and CD8 T cells in the peripheral blood (**I**,**J**). ** *p*< 0.01, *** *p* < 0.001, and **** *p* < 0.0001.

CSF1R (CD115) plays a critical role in the growth and differentiation of macrophages [28]. As shown in the feature plots in Figure 7A, *Csfr1* is highly expressed in the ncMono population and shows a continuous increase during leukemia progression. These observations are confirmed by the violin plots in Figure 7B. Flow cytometric analysis of the GFP-negative cells shows an increase in CD115+ cells in the PB of leukemic mice and an increased fluorescence intensity in these cells during leukemia progression (Figure 7C, Supplementary Figure S5). Flow cytometric analysis (Figure 7D) further demonstrates that *Csfr1* expression is exclusively expressed on the GFP− immune cells but not in the GFP+ cells (Figure 7E).We therefore used the GW2580 CSF1R inhibitor [29], which has been shown to suppress the survival, proliferation, differentiation, and function of macrophages, to determine whether the leukemia-induced immune suppressive macrophages have an effect on survival in vivo. When BBC2 leukemia cells were treated with GW2580 in vitro (Figure 7F), there was only a minor decrease in cell viability consistent with the low level of *Csfr1* expression in the leukemic cells. Leukemic mice (n = 5) were treated daily with 80 mg/kg GW2580 via i.p. injection starting from day 5 for 9 days. Following GW2580 treatment, there is a significant increase in survival in the drug-treated cohort compared with the vehicle-treated cohort (Figure 7G). This increased survival is associated with reduced levels of white blood cell counts and a significant decrease in both the spleen and liver weights (Figure 7H). Flow cytometry demonstrates that the presence of GFP+ leukemia cells was significantly reduced after treatment with GW2580 (Figure 7I,J). The gating of CD11b+F4/80+ cells from GFP−Ly6G− cells confirmed the efficient reduction in macrophages in the treatment group. Accompanied by macrophage depletion, there were significant increases in CD4+ and CD8+ T cells in the GW2580-treated mice, further confirming the macrophage-mediated suppression of immune effector cells. These data indicate a leukemia-promoting role for leukemia-induced macrophages in the animal model.

## 4. Discussion

Macrophages constitute a heterogeneous population of myeloid cells of the innate immune system and are involved in diverse processes in normal physiology as well as in pathological conditions, including cancer [30]. These macrophages are referred to as tumor-associated macrophages (TAMs), which are a subgroup of immune cells present in high numbers in the microenvironment of solid tumors and play important roles in tumorigenesis. The majority of studies involving TAMs, however, have focused on analyses in solid tumors, although there have been some studies describing macrophage function in the bone marrow of AML and ALL patients [31,32,33]. Of note, research to date has focused on tissue-resident or infiltrating macrophages in both solid tumors and leukemias. Most tissue-resident macrophages arise from embryonic precursors that are recruited to the tissues before birth and can be maintained locally, independently of circulating monocyte precursors. There is, however, a paucity of reports of circulating macrophages. Recent studies involving macrophages in leukemias were limited to their presence in the bone marrow and spleens of leukemic patients and mice [34], and their function in supporting the expansion of acute myeloid leukemia cell lines. Other studies [35] investigating the regulation of M1 macrophages focused on macrophage cell lines or bone marrow-derived macrophages in vitro. Here, we provide an in-depth profiling of leukemia-induced circulatory macrophages using both single-cell proteomic and transcriptomic technologies to define the leukemia-induced macrophages and demonstrate that macrophages are a major cell type in the peripheral circulation of leukemic mice. We have also identified potential master regulator genes potentially driving the transition of circulating monocytes into macrophages. These leukemia-induced, macrophage-like ncMonos do not express marker genes for cMonos, such as *Ccr2* and *Ly6c2* (Figure 1C) but express macrophage markers *Csf1r*, *Fcgr4*, *Adgre1* (F4/80), and *Adgre4* [36,37,38], as well as the *Nr4a1* and *Cx3cr1* markers for ncMonos [39,40,41,42].

Our previous studies demonstrated increased levels of MDSC during leukemia development [13]. MDSCs are a heterogeneous group of immune cells that play a critical role in immune suppression. The relationship between M-MDSCs and tumor-associated macrophages (TAMs) has been summarized previously [43], where M-MDSCs are defined as CD11b+Ly6C^Hi^Ly6G− in tumor conditions. M-MDSCs give rise to TAMs in the tumor microenvironment with the reduced expression of Ly6C but increased expression of F4/80 and CD115. As shown in Figure 3, almost all the M-MDSCs (R1, CD11b+Ly6C^Hi^Ly6G−) have activated F4/80 at day 14 and approximately one third of the cells in the R2 CD11b+Ly6C^Int^Ly6G− and R3 CD11b+Ly6C^Low^Ly6G− subgroups show activated macrophage markers. These observations support a transition of monocytes, including M-MDSCs, into macrophage-like ncMono during leukemia progression. While all the CD11b+Ly6G- monocytes contribute to the macrophage pool to different degrees, the majority (>60%) come from the CD11b+Ly6C^Low^ population. This observation is further confirmed by the scRNA-Seq data, where trajectory analysis predicts a transition from cMono to ncMono during leukemia progression, and these leukemia-induced ncMonos co-express macrophage markers (Figure 4). Our studies, therefore, demonstrate the presence of leukemia-induced circulatory macrophages and also provide evidence showing their differentiation from blood monocytes. It is anticipated that the identification of critical modulatory genes associated with this transition process will further enable studies of their development and role in leukemogenesis.

Macrophages are vital tissue components involved in organogenesis, maintaining homeostasis and responses to disease. In a tumor context, TAMs can be designated into two activation states, M1 and M2 [44], due to different polarization in response to various environmental stimuli. M1 macrophages are generally considered tumor-killing macrophages and promote immune responses. In contrast, M2 macrophages, which have a similar phenotype to TAMs, promote tumor growth, angiogenesis, invasion, and metastasis [30], as well as suppress the T-cell-mediated anti-tumor immune response [45]. In the SCLL mouse model, the high level of circulating macrophages is associated with immune suppression and leukemia promotion and therefore represents M2 macrophages. This conclusion is supported by the increased expression of *Erg2* in circulating macrophages, which is reported to be an M2 macrophage marker [46]. *Egr2* is a transcription factor induced by STAT6, IL-4, and IL-13, which regulates the IL-4-induced polarization of murine macrophages [46]. Intriguingly, these leukemia-induced macrophages also show the activation of IL4 and IL13, indicating potential positive feedback for M2 polarization. Also consistent with an M2 phenotype, genes related to immune suppression such as *Nos1*, *Nox1*, and *Cd274* are upregulated in these circulatory macrophages.

In a broader context, several other genetic changes seen in the circulatory macrophages in our SCLL model appear related to macrophage differentiation. For example, the aryl hydrocarbon receptor (*Ahr*) shows a reduced expression compared with naive controls. It has been reported that AHR activation promotes the differentiation and function of monocyte dendritic cells [47] and inhibits monocyte–macrophage differentiation [48,49]. We also observed the upregulation of *Pparg*, a nuclear receptor transcription factor involved in macrophage function and differentiation, which suppresses the inflammatory M1 state and promotes the M2 state of macrophages [50]. *Egr2* was critical for the expression of transcription factors *Cebpβ* and *Pparg* in M2 macrophages. Of the many functions of macrophages, they can also suppress the function of T cells through cell–cell contact. Upregulated genes in the ncMono/macrophage that modulate T-cell activation and function include *Cblb* and *Cd300e*, providing a coordinated suppression of T cells, as seen in leukemic mice. Importantly, our in vitro T-cell suppression assay revealed a remarkable suppression of T-cell proliferation by these leukemia-induced macrophages, and the depletion of these macrophages attenuates leukemia progression in a preclinical animal model. Since *Csfr1*, *F4/80*, *Cd274*, *Cd300e*, and *Pparg* expression is conserved in human macrophages and could be used to detect the presence of leukemia-promoting macrophages directly in leukemia patients.

Maturation from immature cells involves genetic reprogramming in response to triggers from extracellular signals and typically involves an exit from the cell cycle, accompanied by the inactivation of genes supporting cell growth. The transition from cMonos to ncMonos involves the inactivation of members of ribosomal protein families (Rpl and Rps). These proteins are highly expressed in proliferating cells as the demand for proteins increases [51]. In fact, in subgroup 3 from the pseudo-time analysis, 70% of inactivated genes during ncMono maturation are members of these two families. Changes in gene expression are orchestrated by transcription factors. Silencing of the *CD135* (*Flt3*) gene, which is a receptor tyrosine kinase expressed on the surface of most hematopoietic cell precursors, is a key event during macrophage maturation in our SCLL model. In addition, transcription factors such as *Pau2f2* and signaling intermediates such as *Stk10*, *Ptprj*, and *Pik3ap1* are also upregulated in mature macrophages.

In summary, we have demonstrated high levels of previously unappreciated circulating macrophages in the peripheral circulation during FGFR1-driven leukemogenesis. These macrophages are derived from monocytes regardless of their expression levels of Ly6C but are predominantly from the Ly6C^low^ non-classical monocyte population. RNA expression profiling and T-cell suppression assays confirmed an immune suppressive role for these leukemia-induced macrophages. Genetic reprogramming during differentiation from classical to non-classical monocytes and macrophages has been demonstrated, and specific genes related to this continuum have been defined including the upregulation of *Csf1r*. With pharmacological targeting of the CSF1R, the macrophage-specific protein leads to improved survival and reduced levels of both tumor-promoting macrophages and leukemic cells in the mouse leukemia model, highlighting the value of targeting the immune suppressive microenvironment.

## Data Availability

All data generated or analyzed during this study are included in this published article (and its Supplementary Information Files). Further details can be obtained on request. Sequencing data is available in the GEO database, accession number GSE289082.

## Supplementary Materials

The following supporting information can be downloaded at: https://www.mdpi.com/article/10.3390/cells14191533/s1, Figure S1: Violin plots show the relative expression levels of the 39 CyTOF markers across the 15 different clusters; Figure S2: Volcano plots for genes expressed in c-Monos at D11 (A) and D14 (B) compared with naive mice show the increases in high-level gene expression during leukemia progression based on both *p*-value adjusted (*p*adj) and log_2_ fold changes. A comparison of the relative levels of expression of specific genes between naive and D11 and D14 leukemic mice (C). These expression changes for the most highly upregulated and downregulated genes are further defined in the violin plots shown in (D); Figure S3: Comparison of dysregulated pathways between nc-monos and c-monos during leukemia progression (A) displayed as violin plots for selected pathways (B); Figure S4: K means analysis of expression changes (on a log scale) for representative genes during the transition from cMonos to ncMonos; Figure S5: Flow cytometry data shows an increase in the CD115+CD11b+ cells in the GFP− population during leukemia progression. The expression levels of CD115 also increase during the same period; Table S1: The antibody list for CyTOF; and Table S2: Primer sequences used for real-time quantitative PCR (qRT-PCR) analysis. Table S3. Markers for the classical and non-classical monocytes.
