# Supplementary figures and images for: Genomic Analysis Defines Increased Circulating, Leukemia-Induced Macrophages That Promote Immune Suppression in Mouse Models of FGFR1-Driven Leukemogenesis
Source: Cells. 2025 Sep 30;14(19):1533. doi: 10.3390/cells14191533 (PMC12523281; doi:10.3390/cells14191533)

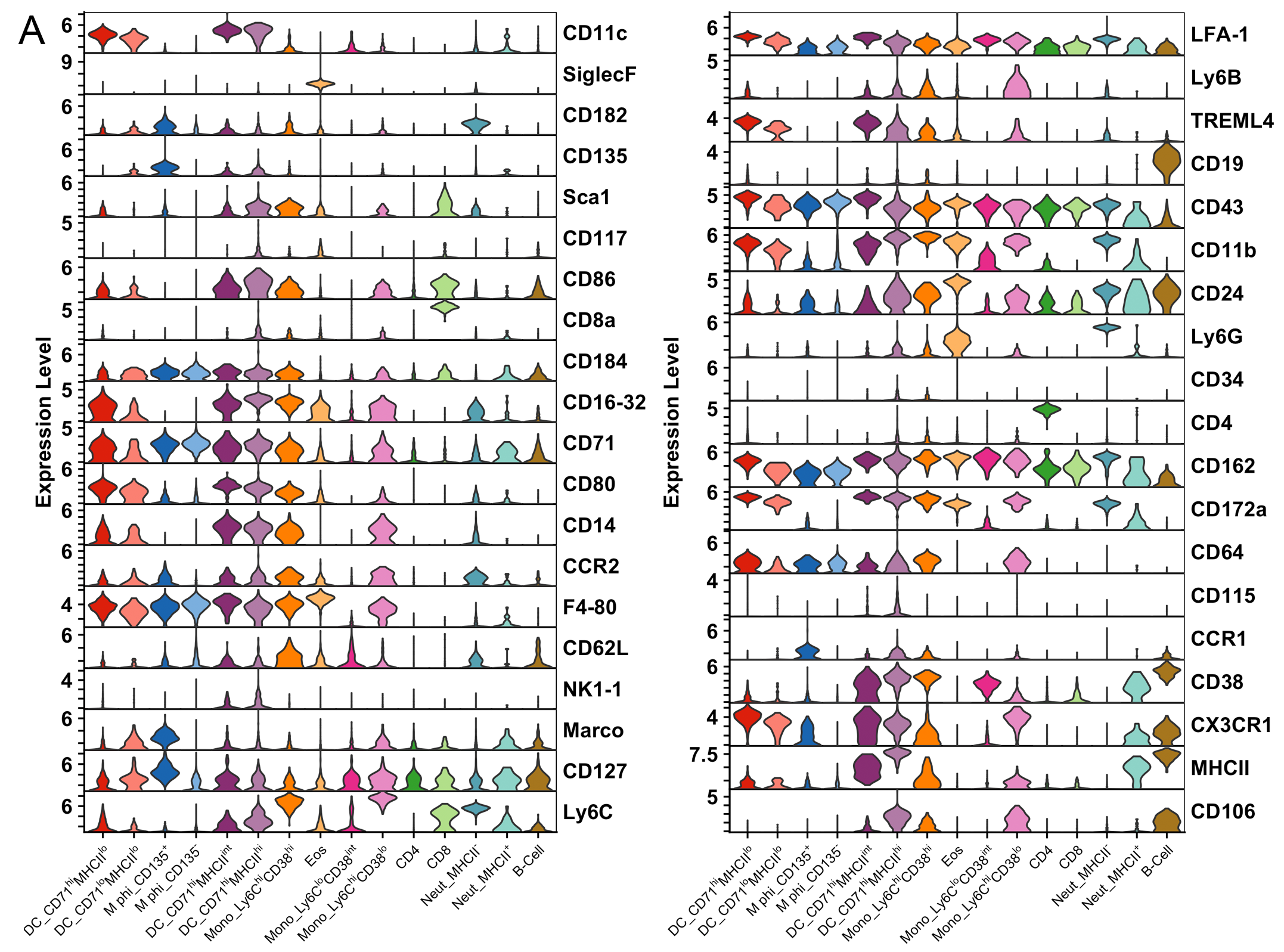

Supplement: Supplementary file 1 [file cells-14-01533-s001.zip › Supplemental Figure S1.tif]

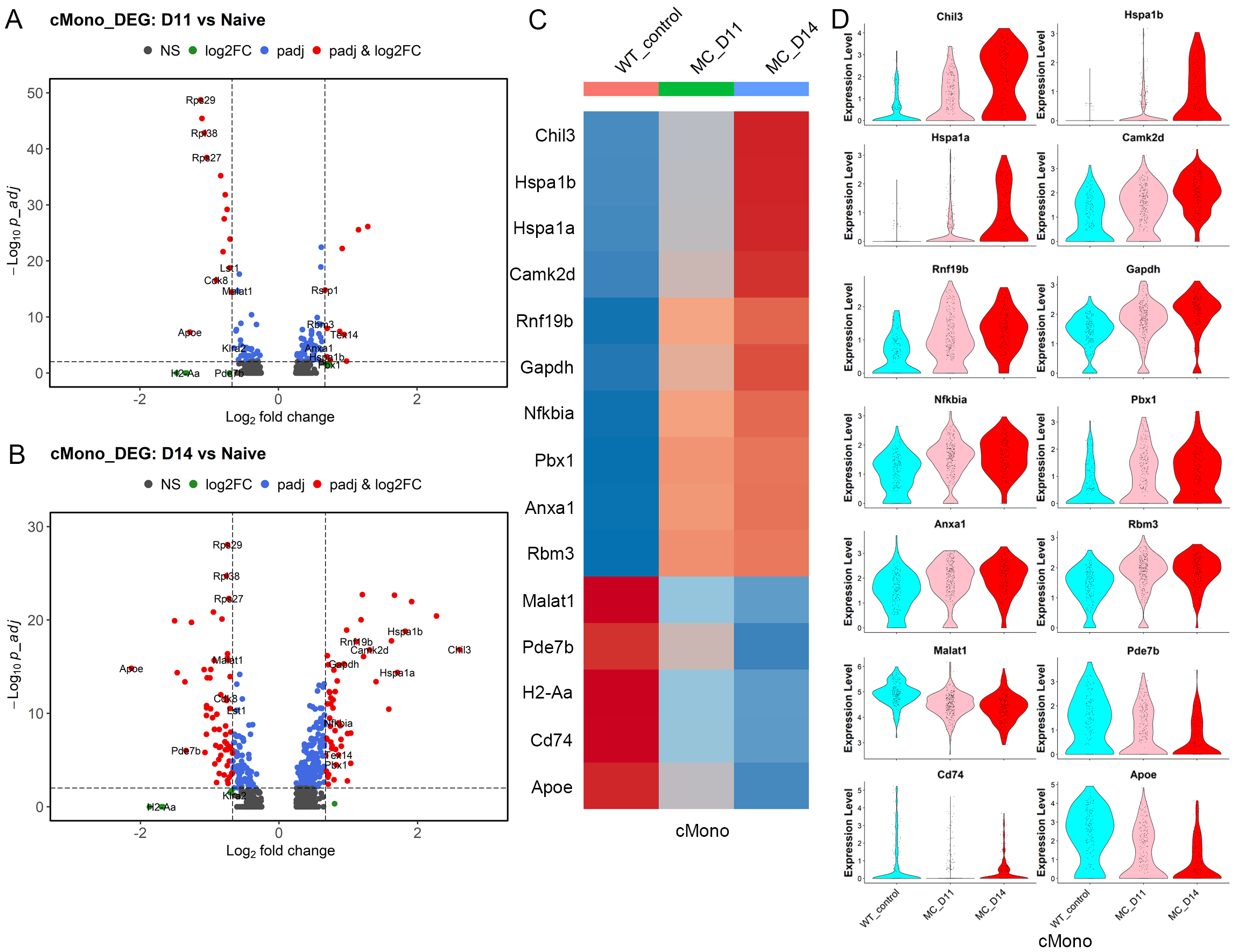

Supplement: Supplementary file 1 [file cells-14-01533-s001.zip › Supplemental Figure S2.tif]

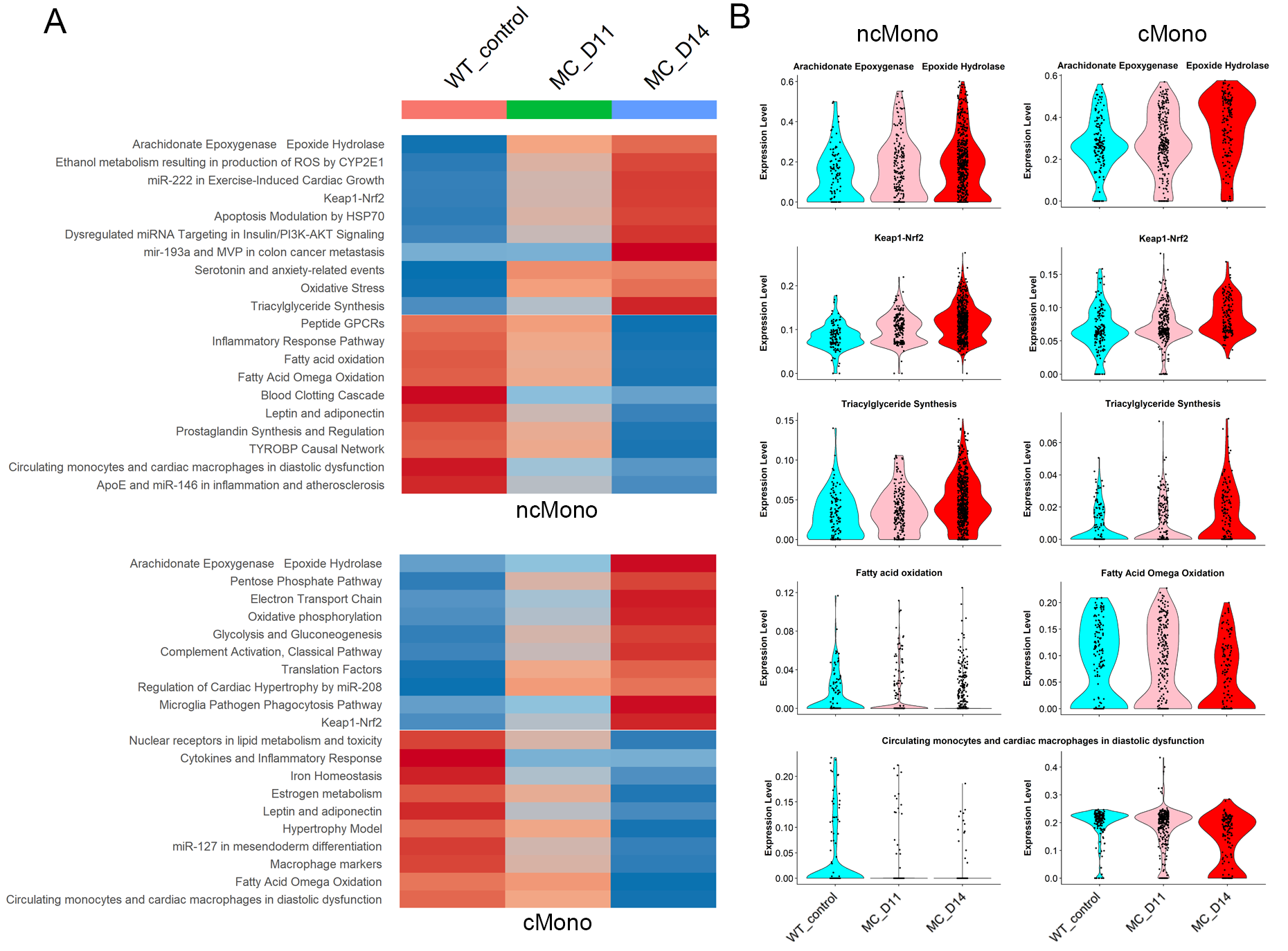

Supplement: Supplementary file 1 [file cells-14-01533-s001.zip › Supplemental Figure S3.tif]

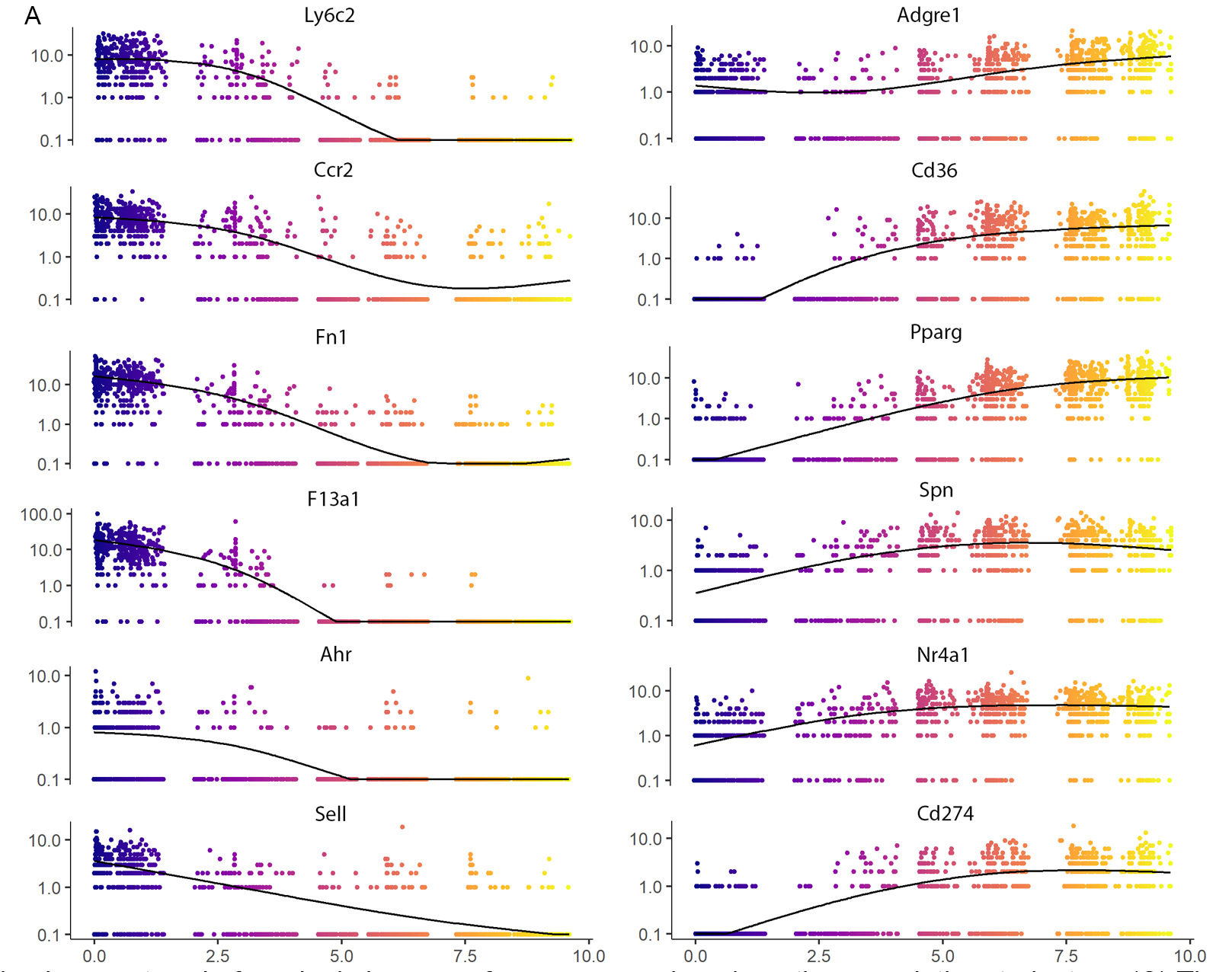

Supplement: Supplementary file 1 [file cells-14-01533-s001.zip › Supplemental Figure S4.tif]

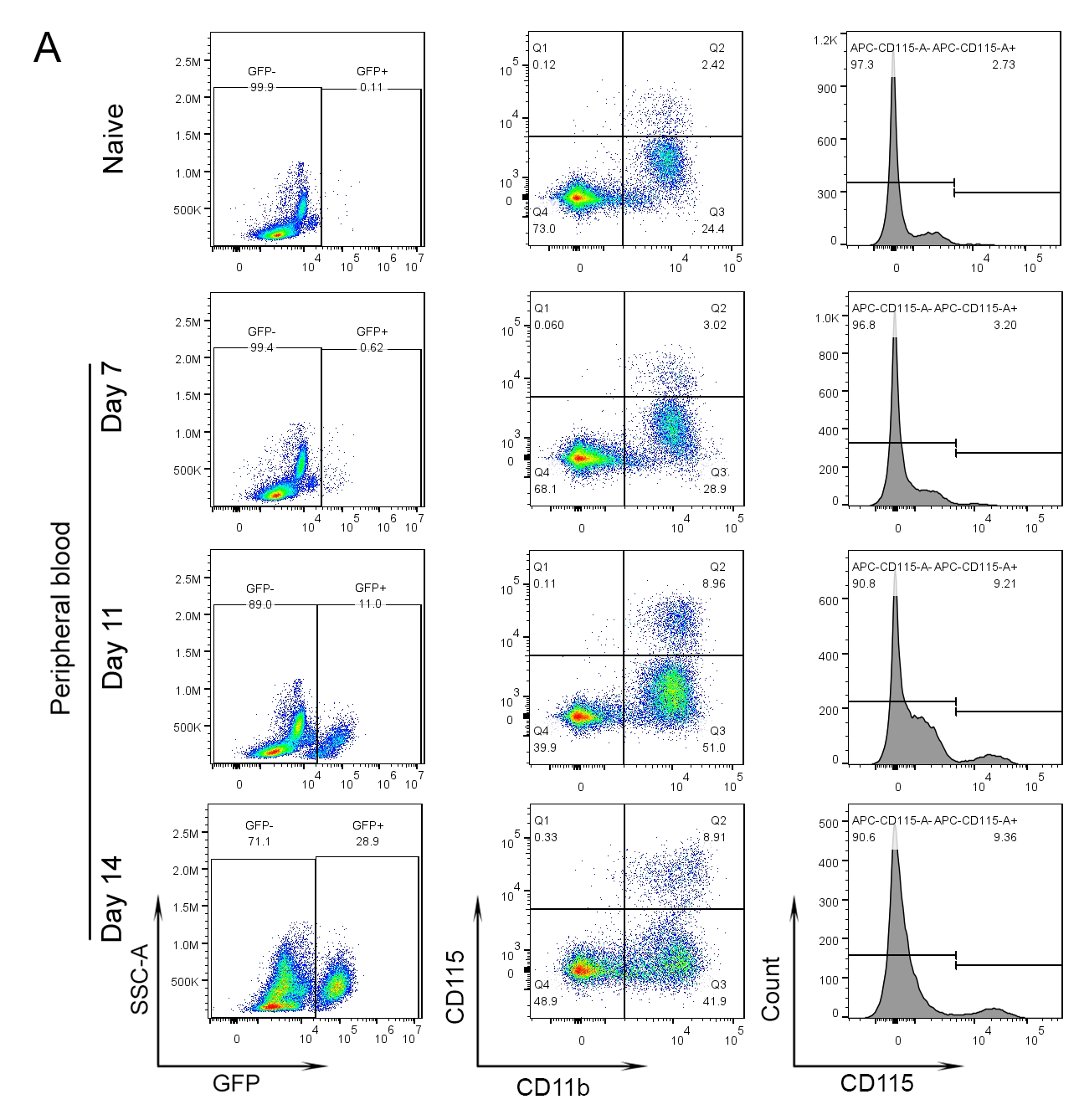

Supplement: Supplementary file 1 [file cells-14-01533-s001.zip › Supplemental Figure S5.tif]
